# Analysis of Activated Platelet-Derived Growth Factor **β** Receptor and Ras-MAP Kinase Pathway in Equine Sarcoid Fibroblasts

**DOI:** 10.1155/2013/283985

**Published:** 2013-07-11

**Authors:** Gennaro Altamura, Annunziata Corteggio, Lubna Nasir, Zheng Qiang Yuan, Franco Roperto, Giuseppe Borzacchiello

**Affiliations:** ^1^Department of Veterinary Medicine and Animal Production, University of Naples Federico II, Via Veterinaria, 180137 Napoli, Italy; ^2^MRC-University of Glasgow Centre for Virus Research, Institute of Infection, Immunity and Inflammation, College of Medical, Veterinary and Life Sciences, Bearsden Road, Glasgow G61 1QH, UK; ^3^University College London, Internal Medicine, Division of Medicine, Faculty of Medical Sciences, Gower Street, London WC1E 6BT, UK

## Abstract

Equine sarcoids are skin tumours of fibroblastic origin affecting equids worldwide. Bovine papillomavirus type-1 (BPV-1) and, less commonly, type-2 are recognized as etiological factors of sarcoids. The transforming activity of BPV is related to the functions of its major oncoprotein E5 which binds to the platelet-derived growth factor **β** receptor (PDGF**β**R) causing its phosphorylation and activation. In this study, we demonstrate, by coimmunoprecipitation and immunoblotting, that in equine sarcoid derived cell lines PDGF**β**R is phosphorylated and binds downstream molecules related to Ras-mitogen-activated protein kinase-ERK pathway thus resulting in Ras activation. Imatinib mesylate is a tyrosine kinase receptors inhibitor which selectively inhibits the activation of PDGF**β**R in the treatment of several human and animal cancers. Here we show that imatinib inhibits receptor phosphorylation, and cell viability assays demonstrate that this drug decreases sarcoid fibroblasts viability in a dose-dependent manner. This study contributes to a better understanding of the molecular mechanisms involved in the pathology of sarcoids and paves the way to a new therapeutic approach for the treatment of this common equine skin neoplasm.

## 1. Introduction

Sarcoids are skin tumours of fibroblastic origin affecting equids and are considered to be the most common equine cutaneous neoplasm worldwide. These tumours are locally invasive, nonmetastatic, and very rarely regress. Sarcoids may exist as single or multiple lesions, most frequently arising from sites of previous injuries such as the skin of the head, ventral abdomen, and the paragenital region, with six clinical types recognized: occult, verrucous, nodular, fibroblastic, mixed, and malignant [1]. They are all histologically characterized by proliferation of spindle-shaped dermal fibroblasts forming whorls and by epidermal hyperplasia, hyperkeratosis, and rete peg formation [2, 3]. Although the pathology of this equine neoplasm is not completely understood, a role for bovine papillomavirus type-1 (BPV-1) and, less commonly, BPV-2 infection had been recognized in the etiology of sarcoids [4, 5]. BPV-1 and -2 are oncogenic double-stranded DNA viruses belonging to the genus of Delta papillomaviruses, which are able to infect both epithelial cells and fibroblasts in their natural host [6]. The oncogenic potential of BPV-1/-2 in bovids is known to be related to the expression of viral oncoproteins E5, E6, and E7 [7]; BPV genes have been found to be expressed in sarcoids, suggesting that the viral proteins also play a role in the development of this equine skin neoplasm [8–12].

E5 is the major oncoprotein encoded by BPVs [13]; it is mostly localized in the endomembrane compartments of the Golgi apparatus (GA), endoplasmic reticulum, and plasma membrane of epithelial cells [14]; cytoplasmic and juxtanuclear expression of E5 has also been reported in sarcoid fibroblasts [11].

PDGF*β*R and other tyrosine kinase receptors are involved in human and animal papillomavirus-induced carcinogenesis since their physiological activity is impaired by E5 oncoproteins of PVs [13]. 

The major transforming activity of BPV-1 E5 is due to its ability to specifically bind and activate PDGF*β*R in a ligand independent manner, and, importantly, it does not activate other related receptors [15–18]. E5 binds to PDGF*β*R as a dimer thereby inducing receptor oligomerization, autophosphorylation, and thus activation [19–21]. PDGF*β*R is constitutively activated in transformed mouse cells expressing BPV-1 E5, and binding of BPV-1 E5 to PDGF*β*R induces its activation in mortal human fibroblasts [18, 22]. Importantly, this interaction also takes place *in vivo*, confirming the role of the E5-PDGF*β*R complex in carcinogenesis [15, 23]; additionally, PDGF*β*R has been found to be activated in BPV-E5 positive urinary bladder tumours and equine sarcoids [24, 25]. 

Activated tyrosine kinase receptors can stimulate intracellular-signalling pathways which regulate cell proliferation, such as the Ras-mitogen-activated protein kinase-ERK (Ras-MAPK-ERK) pathway [26, 27]; once phosphorylated, they recruit growth factor receptor bound protein 2 (GRB2) which is constitutively associated with Sos1 protein, a guanine nucleotide exchange factor of the small GTP-ase, Ras. When recruited to plasma membrane together with GRB2, Sos1 catalyses the switch of Ras from inactive GDP bound form (Ras-GDP) to a transductionally active GTP bound form (Ras-GTP). Ras-GTP binds to and stimulates downstream effectors (among these, Raf1), resulting in phosphorylation and activation of MEK and its downstream kinase ERK, which can regulate cell growth or differentiation [28]. In response to PDGF*β*R stimulation, GRB2 facilitates activation of the Ras-MAPK-ERK pathway [27] playing a role in brain cancer development [29]. BPV E5 induces recruitment of GRB2 to activated PDGF*β*R (pPDGF*β*R), contributing to cell transformation *in vitro* [22]; furthermore, pPDGF*β*R recruits GRB2-Sos1 which promote Ras activation in bovine urinary bladder cancer, suggesting a role of this pathway in BPV-induced carcinogenesis [25].

Imatinib mesylate is a tyrosine kinase receptor inhibitor formerly known as STI-571 or Gleevec; this small molecule, derived from 2-phenylaminopyrimidine, binds to the ATP-binding site, thus inhibiting the tyrosine kinase activity of the PDGFR (both *α* and *β*) and the downstream signals, including the Ras-MAPK-ERK cell proliferation pathway [30–32]. Imatinib has been largely studied in human solid tumours, where it has been shown to induce cell growth arrest [33–37]. Furthermore, imatinib selectively inhibits the Bcr-Abl tyrosine kinase in chronic myeloid leukemia and c-kit in several human cancers, resulting in either apoptosis or inhibition of proliferation [30, 31, 38–42]. Gleevec is used in veterinary medicine for the treatment of canine mast cells tumours (MCTs) [43–45] and has been proposed as therapy for several feline neoplasms, including vaccine associated sarcomas (VAS), in which the PDGF*β*R is selectively inhibited by the treatment [46, 47].

The aim of this study was to investigate the activation of PDGF*β*R, the Ras-MAPK-ERK pathway and to further evaluate the effects of imatinib mesylate on PDGF*β*R phosphorylation and cell viability in an equine sarcoid disease model. 

## 2. Methods

### 2.1. Cells and Cell Culture

Equine sarcoid-derived cell lines EqS01 and EqS04b, expressing endogenous BPV-1 E5, EqS02a transfected with BPV-1 E5 (EqS02aE5) and EqS02a cells which do not express E5 and are not fully transformed, have all been described previously [48, 49]. Cells were cultured in Dulbecco's modified eagle medium (DMEM) supplemented with 10% fetal bovine serum (FBS) (Gibco) or in serum-deprived DMEM depending on the experiment, in a 37°C humidified atmosphere of 5% CO_2_ in air.

### 2.2. Coimmunoprecipitation, Immunoprecipitation, and Immunoblotting

To prepare protein extracts for immunoprecipitation, 80% confluent monolayers in 150 mm dishes were washed three times in phosphate-buffered saline (PBS; pH 7.4, 0.1 M) and then lysed in ice-cold JS buffer (50 mM HEPES, PH 7.5, 150 mM NaCl, 1% glycerol, 1% Triton-X100, 150 *μ*M MgCl_2_, and 5 mM ethylene glycol tetra acetic acid) added with 20 mM sodium pyrophosphate, 0,1 mg mL^−1^ aprotinin, 2 mM phenylmethylsulfonyl fluoride, 10 mM disodium orthovanadate, and 50 mM sodium fluoride. Protein concentrations were determined by use of a protein assay kit (Bio-Rad Laboratories). Equal amounts of protein lysates (500 *μ*g) were immunoprecipitated with 2 *μ*g mL^−1^ rabbit anti-pPDGF*β*R antibody (Santa Cruz Biotechnology) overnight at 4°C. A-G/plus sepharose beads (Santa Cruz Biotechnology) (20 *μ*L) were added to the samples, and the mixture was rotated for 1 h at 4°C. After 3 washings in lysis buffer, the immunoprecipitates were resuspended in Laemmli sample buffer [50] (sodium dodecyl sulphate (SDS), Tris-HCl pH 6.8, glycerol, bromophenol blue, and 2*β*-mercaptoethanol) and analyzed by SDS polyacrylamide gel electrophoresis (PAGE) and immunoblotting. Nitrocellulose membranes were blocked with 5% bovine serum albumin (BSA)-in-Tris-buffered saline (TBS: 12.5 mM TrisHCl pH 7.4; 125 mM NaCl) at room temperature (RT) and incubated O/N at 4°C with rabbit anti-pPDGF*β*R, mouse anti-GRB2 (Upstate Biotechnology) and rabbit anti-Sos1 (Santa Cruz Biotechnology) antibodies diluted 1 : 500, 1 : 1000, and 1 : 200, respectively. After 3 washing steps in TBS-Tween 0.1%, appropriate peroxidase-conjugated secondary antibodies (Amersham, Gel Health Care) were applied 1 h at RT at 1 : 1000 dilution. Membranes were washed again, and bound antibodies were visualized by enhanced chemiluminescence (ECL) (Western Blot Luminol Reagent, Santa Cruz Biotechnology). Protein levels were quantitatively estimated by densitometry using ChemiDoc gel scanner (Bio-Rad Laboratories) equipped with a densitometric workstation (Image Lab software, Bio Rad Laboratories).

Similarly, protein extracts were immunoprecipitated for Sos1 (2 *μ*g mL^−1^) and analyzed by SDS-PAGE and immunoblotting with anti-GRB2 and anti-Sos1 antibodies. The reciprocal coimmunoprecipitation assay could not be performed since the anti-GRB2 antibody does not work for immunoprecipitation.

For evaluation of phosphorylation status of PDGF*β*R in starved serum-deprived cells, the same amount of protein lysates were immunoprecipitated with a rabbit anti-PDGF*β*R antibody (2 *μ*g mL^−1^) (Santa Cruz Biotechnology) and probed for PDGF*β*R presence, using the aforementioned antibody; the membranes were stripped and reprobed with a mouse antiphosphotyrosine (pTyr) antibody at 1 : 1000 dilution (Upstate Biotechnology). Bound antibodies were visualized as mention above.

For the evaluation of activation status of PDGF*β*R after treatment with imatinib mesylate, cell lysates were subjected to immunoprecipitation with rabbit anti-PDGF*β*R and immunoblotting. The membranes were incubated with mouse anti-pTyr antibody, washed, and probed with appropriate secondary antibody. After stripping, rabbit anti-PDGF*β*R antibody was applied and revealed as mentioned above.

Equal volumes (30 *μ*L) of cell lysates were analyzed by immunoblotting for actin levels detection (see the following for details) before coimmunoprecipitation and immunoprecipitation assays to ensure equal amounts of protein loading and allow normalization.

### 2.3. Ras-Pull-Down Assay

To assess Ras activation status, a Ras-pull-down assay (Millipore) was performed according to manufacturer protocol with slight modifications. Briefly, cell lysates were incubated with GST-Raf1-RBD agarose beads for 15 minutes at 4°C with gentle agitation. Beads containing activated Ras were collected by centrifugation, washed with JS lysis buffer, and then prepared and analyzed for immunoblotting with a mouse monoclonal anti-Ras antibody provided by the kit. Raf1-RBD-Ras levels were normalized to total Ras levels and expressed as densitometric ratio. Total Ras expression levels were normalised to actin levels.

### 2.4. Immunoblotting on Whole Cell Lysates

For protein expression analysis on whole cell lysates, equal amounts of proteins were boiled in 2x Laemmli sample buffer [50], electrophoresed, and subjected to immunoblotting; after blocking in TBS-BSA 5%, rabbit anti-PDGF*β*R diluted 1 : 500, rabbit anti-pMEK antibody diluted 1 : 1000, and rabbit anti-pERK (pERK) antibody at 1 : 2000 dilution (Cell Signaling Technology) were applied O/N at 4°C. The membranes were washed and incubated with peroxidase-conjugated anti-rabbit IgG diluted 1 : 1000, 1 h at RT. Following further washing, bound antibodies were visualized as above. The membranes were stripped and reprobed with mouse anti-actin antibody (Calbiochem) at 1 : 5000 dilution to ensure equal amount of proteins for each sample. The protein concentrations were normalised to the actin levels.

### 2.5. RT-PCR and Sequencing of Exons 11–20 of PDGF*β*R Gene

Total RNA from 80% confluent cell monolayers in 60 mm dishes was extracted using RNeasy mini Kit (Qiagen). Following DNase I treatment, first-strand cDNA from PDGF*β*R gene was synthesized using SuperScript III First-Strand Synthesis System for RT-PCR (Invitrogen) according to manufacturer's protocol. cDNA was amplified using different primers sets, which amplified different regions of all sequences from exon 11 to exon 20. The primers sets, the annealing position, the size of amplified fragments, and the annealing temperatures are summarized in Table 1. PCR conditions were as follows: denaturation at 94°C for 20 s, followed by 28 cycles at 94°C for 20 s, 52°C for 30 s, and 68°C for 20 s, with a final extension at 68°C for 10 min. PCR products were separated by electrophoresis in 1% agarose gels with Tris borate ethylene diamine tetra acetic acid (EDTA) buffer (TBE; 89 mMTris base, 89 mM Boric acid, and 2 mM EDTA), stained with ethidium bromide, and visualised under ultraviolet light. Amplicons were purified using the Charge Switch PCR Clean-Up Kit (Invitrogen) following manufacturer's instructions. Cycle sequencing reactions were performed using Applied Biosystems BigDye Terminator Ready Reaction v3.1 Kit, followed by purification of sequence reactions and electrophoresing using ABI Prism 3130XL Genetic Analyser (Applied Biosystems). The sequences were aligned to *Equus caballus *wild type PDGF*β*R using Basic Local Alignment Search Tool (NCBI/BLAST).

### 2.6. Double Immunofluorescence and Confocal Microscopy

EqS cell lines were grown for 2 days on coverslips, washed with PBS, fixed in 4% paraformaldehyde for 20 min, and permeabilized with 0,1% triton X-100 in PBS 5 min. The slides were blocked with 2% BSA for 30 min. Sheep anti-E5 (a kind gift of Professor Maria Saveria Campo, University of Glasgow, Scotland [51, 52]) and rabbit anti-PDGF*β*R primary antibodies were applied O/N at 4°C in a humidified chamber at 1 : 50 dilution in PBS. The slides were washed three times with PBS, then incubated with Alexa Fluor 488 donkey anti-sheep and Alexa Fluor 546 goat anti-rabbit 30 min at RT (Molecular Probes) in a humidified chamber at 1 : 100 dilution. Finally, after washing with PBS, the slides were mounted in aqueous medium PBS : Glycerol 1 : 1 (Sigma). For scanning and photography, a confocal laser-scanning microscope LSM-510 (Zeiss) was used. Alexa Fluor 488/546 was irradiated at 488 nm and 543 nm and detected with a 505–530 nm and 506–615 nm bandpass filters, respectively.

### 2.7. Imatinib Mesylate, Inhibition of PDGF*β*R Activation, and Cell Viability Assay

Imatinib mesylate was provided by LC laboratories (Woburn, USA). For our studies, 50 mM stock solutions were prepared in DMSO and stored at −80°C. Dilutions of imatinib were made from the stock solutions in serum-free medium.

For the evaluation of activation status of PDGF*β*R after drug treatment, 80% confluent cells monolayers in 60 mm dishes were incubated in serum-free medium for 24 h and treated for 15 min with imatinib at the following concentrations: 0,01 *μ*M, 0,1 *μ*M, 1 *μ*M, 5 *μ*M, with or without 30 ng mL^−1^ of human recombinant PDGF-BB (PDGF*β*R natural ligand) (Sigma). Cells were then lysed and subjected to immunoprecipitation of PDGF*β*R and immunoblotting for pTyr and PDGF*β*R as described previously.

For cell viability assay, cells were plated in 96-well microtiter plates at 10000 cells/well and incubated in DMEM 10% FBS in standard conditions. After 24 h, the plates were washed, and serum-free medium containing various concentrations of imatinib with or without 30 ng mL^−1^ of human recombinant PDGF-BB was added. Each condition was replicated in five wells. Relative viable cell numbers were measured after 72 h using the Cell Titer 96 AQ_ ueous_ one solution assay (Promega), a colorimetric system based on the tetrazolium salt MTS, according to manufacturer's protocol. Absorbance at 490 nm was measured using a Sirio-S Reader (Seac and Radim Diagnostics) and Sirio-S v7.0 software.

## 3. Results

### 3.1. PDGF*β*R Activation and Its Binding to Downstream Molecules in Equine Sarcoid Fibroblasts

**Figure 1:**
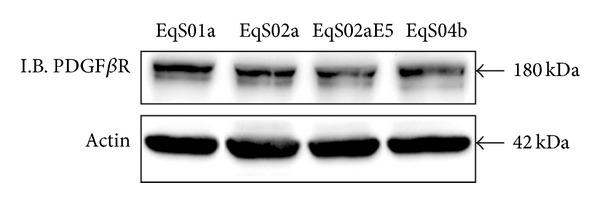
Immunoblotting (I.B.) analysis of PDFG*β*R expression in sarcoid-derived cell lines (EqS). PDFG*β*R was expressed at similar levels in all the analyzed cell lines. Actin protein levels confirmed equal amount of protein loading in each lane.

**Figure 2:**
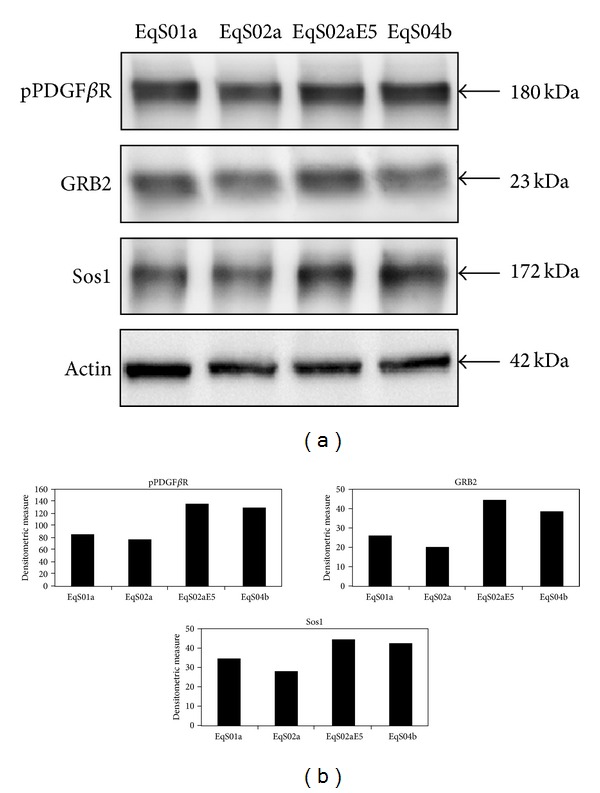
pPDFG*β*R-GRB2-Sos1 coimmunoprecipitation in EqS cell lines. (a) The presence of GRB2 and Sos1 was detected in pPDFG*β*R immunoprecipitates in higher amount in cell lines EqS01a, EqS02aE5, and EqS04b expressing BPV-1 E5 when compared to EqS02a which do not express the oncoprotein. Actin protein levels were detected on whole cell lysates before immunoprecipitation to ensure equal protein loading and allow normalization. (b) Quantitative densitometric analysis of the bands was performed with Image Lab software (ChemiDoc, Bio-Rad Laboratories).

In order to investigate the activation of PDGF*β*R, we first assessed PDGF*β*R expression levels, which were very similar among all EqS cell lines (Figure 1), and then the interaction of activated receptor with its molecular substrates, GRB2 and Sos1. pPDGF*β*R immunoprecipitation followed by immunoblotting for pPDGF*β*R, GRB2, and Sos1-yielded the following results: pPDGF*β*R is immunoprecipitated in higher amounts in EqS02aE5 and EqS04b, both expressing higher levels of BPV-1 E5, and, to a lesser extent, in EqS01a, which expresses lower levels of the oncogene, when compared to EqS02a; the latter cell line harbours very few copies of viral genome, and E5 expression is not detectable [48, 49]; the levels of GRB2 and Sos1 co-immunoprecipitated with pPDGF*β*R were increased in EqS01a, EqS02aE5, and EqS04b correlating with pPDGF*β*R levels, when compared to EqS02a (Figure 2).

**Figure 3:**
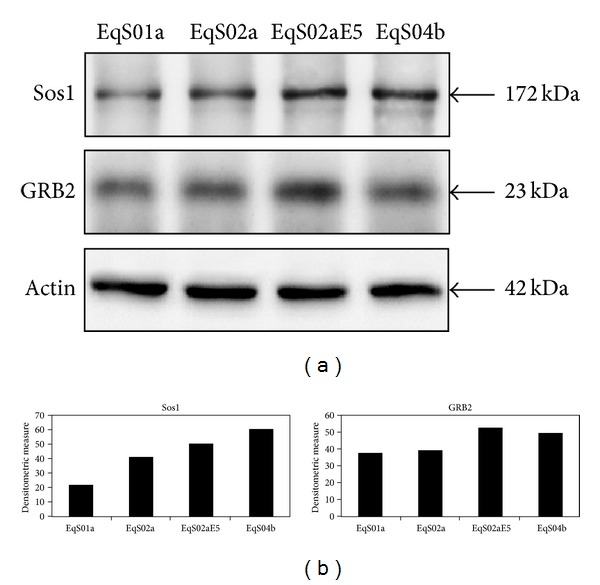
Sos1-GRB2 coimmunoprecipitation. (a) Binding of GRB2 to Sos1 was recorded in all EqS cell lines; the complex was coimmunoprecipitated in higher amount in EqS02aE5 and EqS04b, which express the highest levels of BPV-1 E5 among the analyzed cell lines. Actin protein levels were detected on whole cell lysates before immunoprecipitation to ensure equal protein loading and allow normalization. (b) Quantitative densitometric analysis of the bands was performed with Image Lab software (ChemiDoc, Bio-Rad Laboratories).

Since Sos1 could be recruited to activated PDGF*β*R via GRB2, we next analyzed the physical interaction between Sos1 and GRB2 by coimmunoprecipitation. GRB2 bound Sos1 in all the analyzed cell lines; however, the complex was found in larger amounts in EqS02aE5 and EqS04b (both highly expressing E5) (Figure 3).

### 3.2. Molecular Analysis of Ras-MAPK-ERK Pathway

**Figure 4:**
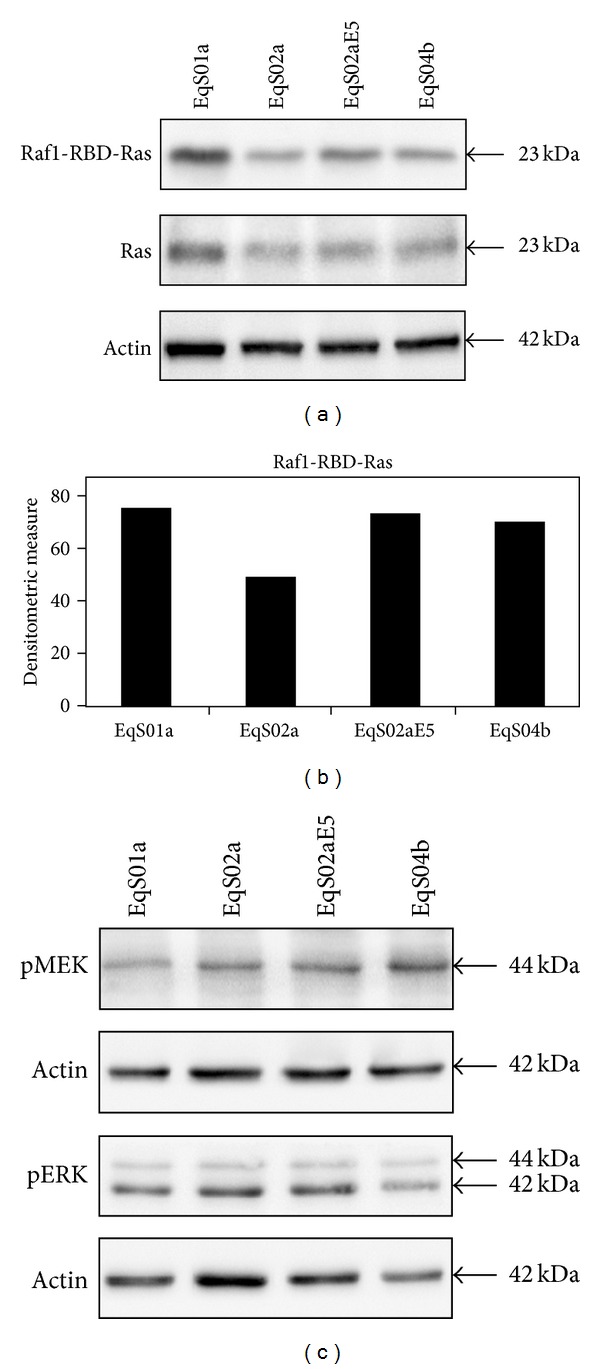
Analysis of Ras-mitogen-activated protein kinase-ERK pathway in sarcoid-derived cell lines (EqS). (a) Immunoblotting analysis of Ras in a pull-down assay of activated Ras (Raf1-RBD-Ras). Raf1-RBD-Ras was increased in cell lines EqS01a (to higher extent), EqS02aE5, and EqS04b when compared to EqS02a (not expressing E5). Total Ras levels were detected on whole cell lysates before pull-down assay to allow normalization of Raf1-RBD-Ras. Ras was overexpressed in EqS01a. Ras expression levels were normalized to actin levels. (b) Quantitative densitometric analysis of the bands was performed with Image Lab software (ChemiDoc, Bio-Rad Laboratories). (c) Immunoblotting analysis of pMEK and pERK expression. No differences in phosphorylation status of both MEK and ERK kinases were recorded. Actin protein levels were detected to ensure equal amounts of proteins for each lane.

To further analyze the downstream signalling molecules of pPDGF*β*R complexed with GRB2-Sos1, we investigated the activation of Ras using a pull-down assay. Firstly, immunoblotting on whole cell lysates collected before performing the pull-down assay showed similar Ras-expression levels in EqS02a, EqS02aE5 and EqS04b. Ras was found to be overexpressed and, consistently, activated at higher levels in EqS01a when compared to other cell lines. Raf1-RBD-Ras was also detected at higher levels in EqS02aE5 and EqS04b when compared to EqS02a (Figures 4(a) and 4(b)). 

The phosphorylation status of ERK (pERK) and its upstream kinase MEK (pMEK) were also determined by immunoblotting using phosphospecific antibodies; however, no differences in expression levels were observed in EqS cell lines (Figure 4(c)).

### 3.3. Analysis of PDGF*β*R Activation in Serum-Starved EqS Cells

**Figure 5:**
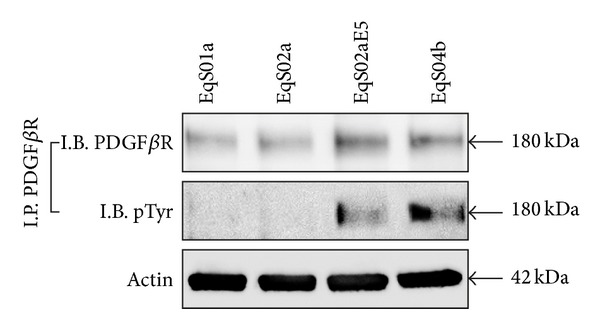
Activation of PDFG*β*R in serum-starved sarcoid-derived cell lines (EqS). The PDFG*β*R immunoprecipitated in serum-deprived cells is phosphorylated only in EqS02aE5 and EqS04b, which express the higher levels of BPV-1 E5 among the analyzed cell lines (I.P.: immunoprecipitation; I.B.: immunoblotting).

EqS cell lines were grown in serum-deprived medium to exclude the possibility that receptor activation could be due to growth factors from FBS. Immunoprecipitation assay, performed by using an anti-PDGF*β*R antibody followed by immunoblotting with anti-PDGF*β*R and anti-pTyr antibodies, revealed that PDGF*β*R is phosphorylated only in EqS02aE5 and EqS04b, both expressing high levels of E5 (Figure 5). 

### 3.4. Sequence Analysis of Exons 11–20 of PDGF*β*R Gene

To further evaluate whether the sustained activation of PDGF*β*R in EqS cell lines might be caused by activating mutations in the transmembrane and/or cytosolic domain, cDNAs of exons 11–20 of PDGF*β*R were amplified by RT-PCR using different primers sets followed by sequencing. Sequence analysis and alignment with wild type sequence revealed that no mutations occurred in these exons in EqS cell lines (data not shown).

### 3.5. Colocalization of PDGF*β*R with BPV-1 E5

**Figure 6:**
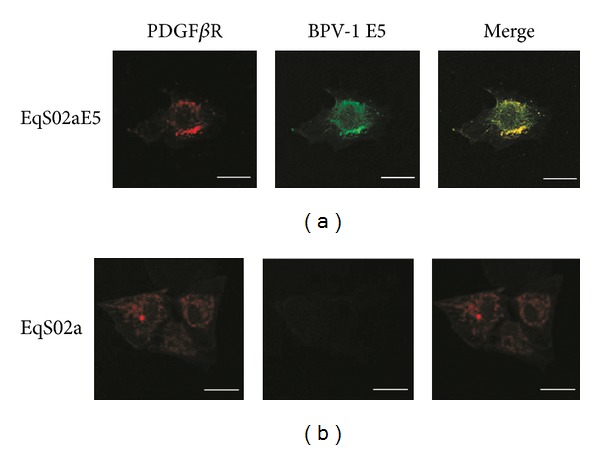
BPV-1E5-PDFG*β*R colocalization. (a) BPV-1 E5 (green fluorescence) and PDFG*β*R (red fluorescence) markedly colocalized (merged panel, yellow fluorescence) in a juxtanuclear Golgi-like position in sarcoid-derived EqS02a cells transfected with BPV-1 E5 (EqS02aE5). A representative image is shown. (b) EqS02a cells expressed PDFG*β*R in the cytoplasm and showed no green signal for E5. Bar = 20 *μ*m.

As gene mutations that may induce activation of PDGF*β*R have been excluded, we next sought to address whether the receptor c-localizes with BPV-1 E5. EqS cell lines were analyzed by double labelling immunofluorescence using rabbit anti-PDGF*β*R and sheep anti-E5 primary antibodies (green fluorescence for E5 and red fluorescence for PDGF*β*R). Interestingly, both proteins appeared to be mostly expressed in a juxtanuclear position in EqS02aE5, where they markedly colocalize as judged by the yellow fluorescence of merged images (Figure 6(a)). PDGF*β*R was found to be expressed also in the cytoplasm of EqS01a, EqS04b (data not shown), and EqS02a cells (Figure 6(b)); the latter cell line showed, as expected, no signal for E5 (Figure 6(b)). BPV-1 E5 staining was not recorded in EqS01a and EqS04b, which is probably due to undetectable expression levels of endogenous protein by immunofluorescence (expression of E5 in these cells has been shown previously by RT-PCR [48]).

### 3.6. Effects of Imatinib Mesylate on PDGF*β*R Phosphorylation and Cell Viability

**Figure 7:**
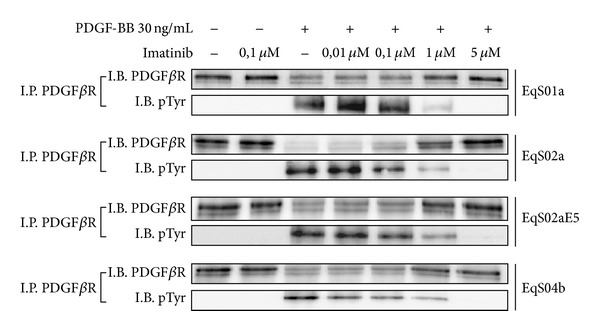
Dose-dependent inhibition of PDGF-BB-induced PDFG*β*R activation by imatinib mesylate. In sarcoid-derived cell lines (EqS) treated with increasing doses of imatinib with or without PDGF-BB, the drug inhibited PDFG*β*R autophosphorylation. PDGF*β*R downregulation was another measure of its activation induced by PDFG-BB, and, conversely, increasing of PDGF*β*R expression in cells treated with higher doses of imatinib confirmed the inhibition of receptor activation (I.P.: immunoprecipitation; I.B.: immunoblotting).

Serum-starved cells were exposed to various concentration of imatinib with or without PDGF-BB in order to determine the effects of the drug on PDGF*β*R activation and cell viability in EqS cell lines. Protein extracts were subjected to immunoprecipitation of PDGF*β*R followed by immunoblotting with anti-pTyr and anti-PDGF*β*R antibodies.  Figure 7 clearly shows that PDGF-BB was able to induce PDGF*β*R transphosphorylation, which indeed was not observed in the absence of ligand; furthermore imatinib is shown to inhibit PDGF*β*R autophosphorylation induced by PDGF-BB in a dose-dependent manner, with near complete inhibition at a concentration of 5 *μ*M in all the cell lines. Additionally, PDGF*β*R expression levels decreased parallel to the increase of its phosphorylation degree, whereas they increased concomitantly with inhibition of phosphorylation by imatinib with a dose-dependent manner. 

**Figure 8:**
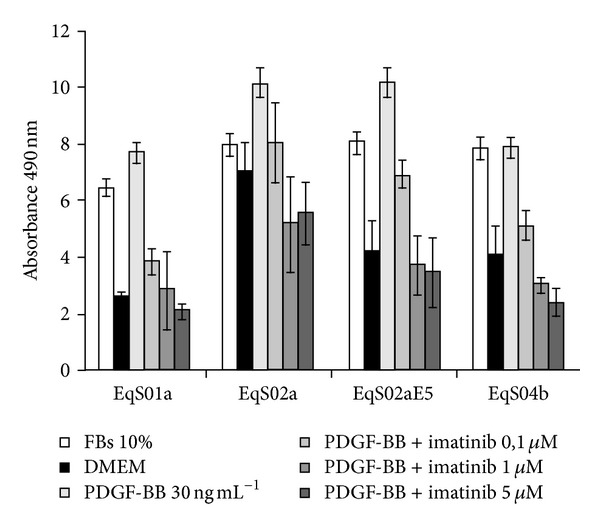
Effects of imatinib mesylate on PDGF-BB-stimulated sarcoids-derived cells (EqS) viability. MTS-based assay revealed that PDGF-BB stimulates cell growth as well as FBS and that imatinib decreases cell viability in all the analyzed cell lines in a dose-dependent manner, but with lower efficiency in EqS02a (not expressing E5). The data represent the mean of repeated independent experiments.

Similarly, cells were exposed to 10% FBS or various concentrations of imatinib with or without PDGF-BB and subjected to MTS-based cell viability assay. As shown in Figure 8, all the cell lines proliferated in the presence of both FBS and PDGF-BB, and imatinib at a concentration of 0.1 *μ*M is already sufficient to severely reduce cell viability in EqS01a, EqS02aE5, and EqS04b; treatment with increasing doses of imatinib caused a further gradual decrease in cell viability levels. All these biological effects appear to be milder in EqS02a, in which no difference in cell viability can be observed at the highest doses of imatinib.

## 4. Discussion

E5 is the major oncoprotein encoded by BPV-1 and plays a key role in the tumorigenic process [13]; the main transforming activity of BPV E5 is due to its specifical binding to PDGF*β*R, causing receptor autophosphorylation and activation, which results in mitogenic signalling and neoplastic transformation [18, 20]. 

Among the downstream pathways of pPDGF*β*R, the Ras-MAPK-ERK signalling is known to regulate cell proliferation [26, 27]. In this study we investigated the activation of PDGF*β*R and Ras-MAPK-ERK pathway in equine sarcoid-derived cell lines EqS01a, EqS02a, EqS02aE5, and EqS04b [48, 49]. We found that PDGF*β*R was phosphorylated and bound its downstream partners GRB2-Sos1 in higher amounts in EqS02aE5 and EqS04b (both containing high levels of oncoproteins transcripts) and, to a lesser extent, in EqS01a when compared to EqS02a (which expresses low and not detectable levels of oncoproteins, resp.). Consistently, active Ras was expressed at higher levels in EqS cell lines expressing E5, suggesting that BPV-1 E5 may contribute to activation of this pathway and lead to sarcoid fibroblasts full transformation. Accordingly, PDGF*β*R was found to be constitutively activated in transformed mouse cells expressing BPV-1 E5, and pPDGF*β*R binds GRB2 in mortal human fibroblasts transfected with the oncoprotein [18, 22]; moreover, PDGF*β*R has been found to be activated *in vivo* in BPV-E5 positive equine sarcoids, and pPDGF*β*R recruits GRB2-Sos1 which enhance Ras activation in bovine urinary bladder cancer [24, 25]; our finding of total Ras overexpression in EqS01a cell line is in agreement with previous studies which reported an association between PV infection in bovids and activation of Ras gene [53]: these data may suggest a role of this pathway in BPV-induced carcinogenesis. In addition, Ras had been found to be activated in cultured fibroblasts also by Rhesus-PV E5, indicating that E5 genes may play a major role in the regulation of this transduction pathway [54].

Surprisingly, no differences were found in phosphorylation status of the downstream kinases of activated Ras, namely, pMEK and pERK. Previous studies showed that E5 does not promote any changes in ERK activity in cultured fibroblasts [22, 55] as well as in BPV-induced tumours *in vivo*: it is therefore possible that pPDGF*β*R activates GRB2, Sos1, and Ras which may deviate on phosphatidylinositol-3-kinase/AKT pathway (PI3 K/AKT). As matter of the fact, PI3 K/AKT pathway, rather than MEK-ERK signalling, had been found to be activated in BPV-induced tumours, thus contributing to neoplastic transformation [25, 56]. 

To further investigate the possible factors contributing to PDGF*β*R activation, we first assessed PDGF*β*R phosphorylation status on serum starved cells: the receptor was found to be phosphorylated only in EqS02aE5 and EqS04b, which express the highest levels of BPV-1 E5 among all cell lines, suggesting that its activation may be due to the interaction with the E5 oncoprotein rather than to the presence of growth factors added to the medium. Accordingly, Petti et al. demonstrated that BPV-1 E5 activates PDGF*β*R in a ligand-independent manner [18]. Taken together, all these findings may indicate that the extent of PDGF*β*R activation may correlate with E5 expression levels and the number of viral copies; direct association of viral load with impaired expression of tyrosine kinase receptors has been reported for human cervical tumours harboring human papillomavirus, suggesting that the impairing of these cellular functions may depend on the viral load in PVs induced cancer [57]. 

PDGFR and other related tyrosine kinase receptors may be activated by mutations or genetic rearrangements causing tumours in both human and animal species, particularly in the transmembrane and cytosolic domains [58–64]. Sequence analysis of PDGF*β*R in our cell lines revealed that no mutations occurred along these domains, suggesting that the receptor is not activated by itself in our experimental model.

Furthermore, double labeling immunofluorescence showed that PDGF*β*R perfectly colocalized with BPV-1 E5 in EqS02aE5 where it was mostly expressed in a juxtanuclear position, consistently with the location of E5 in the Golgi apparatus (GA) [14, 65]. The intracellular colocalization of E5 and PDGF*β*R indicates that this protein interaction may take place in the GA and may be another proof of evidence of the possible receptor activation upon E5 interaction; juxtanuclear colocalization of E5 with PDGF*β*R was also reported in bovine urinary bladder cancer by this research group, suggesting that this finding could be common in BPV-induced tumours [15]. Many studies have reported the physical interaction between these two proteins and subsequent activation of PDGF*β*R both *in vitro* and *in vivo* [23, 66], thus we speculate that BPV-1 E5 may bind to PDGF*β*R also in our sarcoid-derived cell lines expressing E5, inducing its phosphorylation and activation of downstream pathways leading to transformation.

Although many therapeutical strategies have been proposed for treatment of sarcoid, no 100% effective therapy is available so far [1]. Many tyrosine kinase receptors inhibitors are used in veterinary medicine [67]; among these, imatinib mesylate (also known as STI-571 or Gleevec) has been shown to selectively inhibit PDGF*β*R activation in both human and animal tumours [33–36, 47]. In this study we showed that sarcoid fibroblasts proliferation was mostly dependent on PDGF*β*R stimulation; furthermore, we demonstrated that imatinib inhibited PDGF*β*R phosphorylation in a dose-dependent manner. In this case no phosphorylation of PDGF*β*R could be observed in serum-free conditions, whilst the receptor was phosphorylated after 24 h of starvation, suggesting that longer times are needed to reach detectable levels of its activation. The finding of total PDGF*β*R downregulation concomitant to the augmentation of its phosphorylation degree induced by PDGF-BB, was not surprising: for the PDGF*β*R and other tyrosine kinase receptors, ligand binding induces receptor phosphorylation and thus triggers its clustering in coated pits, followed by endocytosis and lysosomal degradation of receptor-ligand complexes [68, 69]. Thus the augmentation of PDGF*β*R expression levels at increasing doses of drug was another measure of the efficiency of treatment. Here we also demonstrated that imatinib was able to decrease sarcoid fibroblasts cell viability in a dose-dependent manner. However, lower effects were observed on cell viability in EqS02a by imatinib treatment at all the experimental doses; lacking E5 expression, this cell line may have lower levels of activated PDGF*β*R, thus the drug may have less access to the ATP-binding site and, as a consequence, milder biological effects when compared to other cell lines. These findings strengthen our hypothesis of a PDGF*β*R activation by E5 and suggest that imatinib may target more efficiently on fully transformed sarcoid fibroblasts.

## 5. Conclusions

Finally, our study demonstrates that PDGF*β*R is activated thus binding downstream molecular partners in sarcoid-derived cell lines expressing BPV-1 E5; this activation results in Ras activation but not major phosphorylation of MEK and ERK kinases, suggesting that this signalling cascade may possibly cross-talk with other transduction pathways which had been found to be activated in BPV-induced tumours. Further investigations are needed to clarify the specific roles of activated PDGF*β*R and downstream pathways in the pathology of equine sarcoid. Furthermore, the data obtained by treatment of sarcoid-derived fibroblasts with imatinib, suggest that this drug could be proposed for a clinical trial leading to therapy of sarcoid *in vivo*. We have developed a translational approach, and a clinical trial is ongoing to test on sarcoid-affected donkeys the therapeutic effects of a dermatological cream based on Imatinib mesylate (CESA protocol number 2012/0052665).

## Figures and Tables

**Table 1:** Primers sets used to amplify cDNA from exons 11–20 of PDGF**β**R in EqS cells.

Primers sets	Annealing position	Annealing *T*°	Fragments size
F1: ATCCTCATCATGCTCTGGCAG	F1: 2119	52°C	628 bp
R1: TCTCCTTTCATGTCCAGCATG	R1: 2747		
F1: ATCCTCATCATGCTCTGGCAG	F1: 2119	52°C	1226 bp
R3: TCGAGAAGCAGCACCAGCTG	R3: 3344		
F3: TGGCTACATGGACATGAGCAAG	F3: 2682	52°C	663 bp
R3: TCGAGAAGCAGCACCAGCTG	R3: 3344		
F3: TGGCTACATGGACATGAGCAAG	F3: 2682	52°C	593 bp
R2: ATCTCGTAGATCTCGTCGGAG	R2: 3275		

*T*°: temperature; F: forward; R: reverse; bp: base pairs.

## References

[1] Borzacchiello G, Corteggio A (2009). Equine sarcoid: state of the art. *Ippologia*.

[2] Martens A, De Moor A, Demeulemeester J, Ducatelle R (2000). Histopathological characteristics of five clinical types of equine sarcoid. *Research in Veterinary Science*.

[3] Scott DW, Miller WH (2003). Equine dermatology. *Journal of Equine Veterinary Science*.

[4] Lancaster WD, Theilen GH, Olson C (1979). Hybridization of bovine papilloma virus type 1 and type 2 DNA to DNA from virus-induced hamster tumors and naturally occurring equine tumors. *Intervirology*.

[5] Gorman NT (1985). Equine sarcoid—time for optimism. *Equine Veterinary Journal*.

[6] Nasir L, Campo MS (2008). Bovine papillomaviruses: their role in the aetiology of cutaneous tumours of bovids and equids. *Veterinary Dermatology*.

[7] Borzacchiello G, Roperto F (2008). Bovine papillomaviruses, papillomas and cancer in cattle. *Veterinary Research*.

[8] Nasir L, Reid SWJ (1999). Bovine papillomaviral gene expression in equine sarcoid tumours. *Virus Research*.

[9] Carr EA, Théon AP, Madewell BR, Hitchcock ME, Schlegel R, Schiller JT (2001). Expression of a transforming gene (E5) of bovine papillomavirus in sarcoids obtained from horses. *American Journal of Veterinary Research*.

[10] Chambers G, Ellsmore VA, O’Brien PM (2003). Sequence variants of bovine papillomavirus E5 detected in equine sarcoids. *Virus Research*.

[11] Borzacchiello G, Russo V, Della Salda L, Roperto S, Roperto F (2008). Expression of platelet-derived growth factor-beta receptor and bovine papillomavirus E5 and E7 oncoproteins in equine sarcoid. *Journal of Comparative Pathology*.

[12] Corteggio A, Altamura G, Roperto F, Borzacchiello G (2013). Bovine papillomavirus E5 and E7 oncoproteins in naturally occurring tumors: are two better than one?. *Infectious Agents and Cancer*.

[13] Venuti A, Paolini F, Nasir L (2011). Papillomavirus E5: the smallest oncoprotein with many functions. *Molecular Cancer*.

[14] Pennie WD, Grindlay GJ, Cairney M, Campo MS (1993). Analysis of the transforming functions of bovine papillomavirus type 4. *Virology*.

[15] Borzacchiello G, Russo V, Gentile F (2006). Bovine papillomavirus E5 oncoprotein binds to the activated form of the platelet-derived growth factor *β* receptor in naturally occurring bovine urinary bladder tumours. *Oncogene*.

[16] DiMaio D, Mattoon D (2001). Mechanisms of cell transformation by papillomavirus E5 proteins. *Oncogene*.

[17] Goldstein DJ, Li W, Wang L-M (1994). The bovine papillomavirus type 1 E5 transforming protein specifically binds and activates the *β*-type receptor for the platelet-derived growth factor but not other related tyrosine kinase-containing receptors to induce cellular transformation. *Journal of Virology*.

[18] Petti L, Nilson LA, DiMaio D (1991). Activation of the platelet-derived growth factor receptor by the bovine papillomavirus E5 transforming protein. *The EMBO Journal*.

[19] Nappi VM, Petti LM (2002). Multiple transmembrane amino acid requirements suggest a highly specific interaction between the bovine papillomavirus E5 oncoprotein and the platelet-derived growth factor beta receptor. *Journal of Virology*.

[20] Lai C-C, Henningson C, Dimaio D (1998). Bovine papillomavirus E5 protein induces oligomerization and trans-phosphorylation of the platelet-derived growth factor *β* receptor. *Proceedings of the National Academy of Sciences of the United States of America*.

[21] Klein O, Kegler-Ebo D, Su J, Smith S, DiMaio D (1999). The bovine papillomavirus E5 protein requires a juxtamembrane negative charge for activation of the platelet-derived growth factor *β* receptor and transformation of C127 cells. *Journal of Virology*.

[22] Petti LM, Ricciardi EC, Page HJ, Porter KA (2008). Transforming signals resulting from sustained activation of the PDGF*β* receptor in mortal human fibroblasts. *Journal of Cell Science*.

[23] Roperto S, Borzacchiello G, Esposito I (2012). Productive infection of bovine papillomavirus type 2 in the placenta of pregnant cows affected with urinary bladder tumors. *PLoS ONE*.

[24] Borzacchiello G, Mogavero S, De Vita G, Roperto S, Della Salda L, Roperto F (2009). Activated platelet-derived growth factor *β* receptor expression, PI3K-AKT pathway molecular analysis, and transforming signals in equine sarcoids. *Veterinary Pathology*.

[25] Corteggio A, Di Geronimo O, Roperto S, Roperto F, Borzacchiello G (2012). Activated platelet-derived growth factor *β* receptor and Ras-mitogen-activated protein kinase pathway in natural bovine urinary bladder carcinomas. *Veterinary Journal*.

[26] Carnero A (2010). The PKB/AKT pathway in cancer. *Current Pharmaceutical Design*.

[27] Moeller SJ, Head ED, Sheaff RJ (2003). p27Kip1 inhibition of GRB2-SOS formation can regulate Ras activation. *Molecular and Cellular Biology*.

[28] Dance M, Montagner A, Salles J-P, Yart A, Raynal P (2008). The molecular functions of Shp2 in the Ras/Mitogen-activated protein kinase (ERK1/2) pathway. *Cellular Signalling*.

[29] Lokker NA, Sullivan CM, Hollenbach SJ, Israel MA, Giese NA (2002). Platelet-derived growth factor (PDGF) autocrine signaling regulates survival and mitogenic pathways in glioblastoma cells: evidence that the novel PDGF-C and PDGF-D ligands may play a role in the development of brain tumors. *Cancer Research*.

[30] Druker BJ, Tamura S, Buchdunger E (1996). Effects of a selective inhibitor of the Ab1 tyrosine kinase on the growth of Bcr-Ab1 positive cells. *Nature Medicine*.

[31] Buchdunger E, Zimmermann J, Mett H (1996). Inhibition of the Abl protein-tyrosine kinase in vitro and in vivo by a 2- phenylaminopyrimidine derivative. *Cancer Research*.

[32] Buchdunger E, Cioffi CL, Law N (2000). Abl protein-tyrosine kinase inhibitor STI571 inhibits in vitro signal transduction mediated by c-Kit and platelet-derived growth factor receptors. *The Journal of Pharmacology and Experimental Therapeutics*.

[33] Kilic T, Alberta JA, Zdunek PR (2000). Intracranial inhibition of platelet-derived growth factor-mediated glioblastoma cell growth by an orally active kinase inhibitor of the 2-phenylaminopyrimidine class. *Cancer Research*.

[34] Rubin BP, Schuetze SM, Eary JF (2002). Molecular targeting of platelet-derived growth factor B by imatinib mesylate in a patient with metastatic dermatofibrosarcoma protuberans. *Journal of Clinical Oncology*.

[35] Sjöblom T, Shimizu A, O’Brien KP (2001). Growth inhibition of dermatofibrosarcoma protuberans tumors by the platelet-derived growth factor receptor antagonist STI571 through induction of apoptosis. *Cancer Research*.

[36] McGary EC, Weber K, Mills L (2002). Inhibition of platelet-derived growth factor-mediated proliferation of osteosarcoma cells by the novel tyrosine kinase inhibitor STI571. *Clinical Cancer Research*.

[37] Gilbert RE, Kelly DJ, McKay T (2001). PDGF signal transduction inhibition ameliorates experimental mesangial proliferative glomerulonephritis. *Kidney International*.

[38] Druker BJ, Lydon NB (2000). Lessons learned from the development of an Abl tyrosine kinase inhibitor for chronic myelogenous leukemia. *The Journal of Clinical Investigation*.

[39] Mauro MJ, O’Dwyer M, Heinrich MC, Druker BJ (2002). STI571: a paradigm of new agents for cancer therapeutics. *Journal of Clinical Oncology*.

[40] Krystal GW, Honsawek S, Litz J, Buchdunger E (2000). The selective tyrosine kinase inhibitor STI571 inhibits small cell lung cancer growth. *Clinical Cancer Research*.

[41] van Oosterom AT, Judson I, Verweij J (2001). Safety and efficacy of imatinib (STI571) in metastatic gastrointestinal stromal tumours: a phase I study. *The Lancet*.

[42] van Oosterom AT, Judson IR, Verweij J (2002). Update of phase I study of imatinib (STI571) in advanced soft tissue sarcomas and gastrointestinal stromal tumors: a report of the EORTC Soft Tissue and Bone Sarcoma Group. *European Journal of Cancer*.

[43] Isotani M, Ishida N, Tominaga M (2008). Effect of tyrosine kinase inhibition by imatinib mesylate on mast cell tumors in dogs. *Journal of Veterinary Internal Medicine*.

[44] Marconato L, Bettini G, Giacoboni C (2008). Clinicopathological features and outcome for dogs with mast cell tumors and bone marrow involvement. *Journal of Veterinary Internal Medicine*.

[45] Yamada O, Kobayashi M, Sugisaki O (2011). Imatinib elicited a favorable response in a dog with a mast cell tumor carrying a c-kit c.1523A>T mutation via suppression of constitutive KIT activation. *Veterinary Immunology and Immunopathology*.

[46] Lachowicz JL, Post GS, Brodsky E (2005). A phase I clinical trial evaluating imatinib mesylate (Gleevec) in tumor-bearing cats. *Journal of Veterinary Internal Medicine*.

[47] Katayama R, Huelsmeyer MK, Marr AK, Kurzman ID, Thamm DH, Vail DM (2004). Imatinib mesylate inhibits platelet-derived growth factor activity and increases chemosensitivity in feline vaccine-associated sarcoma. *Cancer Chemotherapy and Pharmacology*.

[48] Yuan ZQ, Gault EA, Gobeil P, Nixon C, Campo MS, Nasir L (2008). Establishment and characterization of equine fibroblast cell lines transformed in vivo and in vitro by BPV-1: Model systems for equine sarcoids. *Virology*.

[49] Yuan Z, Gault EA, Saveria Campo M, Nasir L (2011). Different contribution of bovine papillomavirus type 1 oncoproteins to the transformation of equine fibroblasts. *The Journal of General Virology*.

[50] Laemmli UK (1970). Cleavage of structural proteins during the assembly of the head of bacteriophage T4. *Nature*.

[51] Borzacchiello G, Russo V, Spoleto C (2007). Bovine papillomavirus type-2 DNA and expression of E5 and E7 oncoproteins in vascular tumours of the urinary bladder in cattle. *Cancer Letters*.

[52] Silva MA, Altamura G, Corteggio A (2013). Expression of connexin 26 and bovine papillomavirus E5 in cutaneous fibropapillomas of cattle. *The Veterinary Journal*.

[53] Campo MS, McCaffery RE, Doherty I, Kennedy IM, Jarrett WFH (1990). The Harvey ras 1 gene is activated in papillomavirus-associated carcinomas of the upper alimentary canal in cattle. *Oncogene*.

[54] Ghai J, Ostrow RS, Tolar J (1996). The E5 gene product of rhesus papillomavirus is an activator of endogenous Ras and phosphatidylinositol-3′-kinase in NIH 3T3 cells. *Proceedings of the National Academy of Sciences of the United States of America*.

[55] Zago M, Campo MS, O’Brien V (2004). Cyclin A expression and growth in suspension can be uncoupled from p27 deregulation and extracellular signal-regulated kinase activity in cells transformed by bovine papillomavirus type 4 E5. *The Journal of General Virology*.

[56] Corteggio A, Di Geronimo O, Roperto S, Roperto F, Borzacchiello G (2011). Bovine papillomavirus E7 oncoprotein binds to p600 in naturally occurring equine sarcoids. *The Journal of General Virology*.

[57] Song SH, Lee JK, Hur JY, Kim I, Saw HS, Park YK (2006). The expression of epidermal growth factor receptor, vascular endothelial growth factor, matrix metalloproteinase-2, and cyclooxygenase-2 in relation to human papilloma viral load and persistence of human papillomavirus after conization with negative margins. *International Journal of Gynecological Cancer*.

[58] Golub TR, Barker GF, Lovett M, Gary Gilliland D (1994). Fusion of PDGF receptor *β* to a novel ets-like gene, tel, in chronic myelomonocytic leukemia with t(5;12) chromosomal translocation. *Cell*.

[59] Heinrich MC, Corless CL, Duensing A (2003). PDGFRA activating mutations in gastrointestinal stromal tumors. *Science*.

[60] Hirota S, Ohashi A, Nishida T (2003). Gain-of-function mutations of platelet-derived growth factor receptor *α* gene in gastrointestinal stromal tumors. *Gastroenterology*.

[61] Hirota S (2003). Gain-of-function mutation of c-kit gene and molecular target therapy in GISTs. *Japanese Journal of Gastroenterology*.

[62] Sirvent N, Maire G, Pedeutour F (2003). Genetics of dermatofibrosarcoma protuberans family of tumors: from ring chromosomes to tyrosine kinase inhibitor treatment. *Genes Chromosomes and Cancer*.

[63] Giantin M, Vascellari M, Morello EM (2012). c-KIT messenger RNA and protein expression and mutations in canine cutaneous mast cell tumors: correlations with post-surgical prognosis. *Journal of Veterinary Diagnostic Investigation*.

[64] Matsumura I, Mizuki M, Kanakura Y (2008). Roles for deregulated receptor tyrosine kinases and their downstream signaling molecules in hematologic malignancies. *Cancer Science*.

[65] Burkhardt A, Willingham M, Gay C, Jeang K-T, Schlegel R (1989). The E5 oncoprotein of bovine papillomavirus is oriented asymmetrically in Golgi and plasma membranes. *Virology*.

[66] Talbert-Slagle K, DiMaio D (2009). The bovine papillomavirus E5 protein and the PDGF *β* receptor: it takes two to tango. *Virology*.

[67] London CA (2009). Tyrosine kinase inhibitors in veterinary medicine. *Topics in Companion Animal Medicine*.

[68] Nilsson J, Thyberg J, Heldin CH, Westermark B, Wasteson A (1983). Surface binding and internalization of platelet-derived growth factor in human fibroblasts. *Proceedings of the National Academy of Sciences of the United States of America*.

[69] Goldstein JL, Brown MS, Anderson RG, Russell DW, Schneider WJ (1985). Receptor-mediated endocytosis: concepts emerging from the LDL receptor system. *Annual Review of Cell Biology*.

